# Glimpse into the role of Kupffer cells in a spheroid model of metabolic dysfunction-associated steatohepatitis (MASH)

**DOI:** 10.1242/bio.062552

**Published:** 2026-06-03

**Authors:** Niklas Bogovic, Maria Drießlein, Marie Keller, Hans J. Schlitt, Edward K. Geissler, Lydia Schneider, Philipp Kreiner, Henrik Junger, Elke Eggenhofer

**Affiliations:** Department of Surgery, University Hospital Regensburg, 93053 Regensburg, Germany

**Keywords:** 3D liver spheroids, MASH, Steatohepatitis, *In vitro* disease model, Liver fibrosis, Kupffer cells

## Abstract

Metabolic dysfunction-associated steatotic liver disease (MASLD) and its progressive form, metabolic dysfunction-associated steatohepatitis (MASH), are characterized by lipid accumulation, inflammation, and fibrosis. Existing preclinical models often fail to capture multicellular interactions that shape disease progression. In this study, we established a human multicellular 3D liver spheroid model comprising HepaRG hepatocytes, hepatic stellate cells, liver sinusoidal endothelial cells, and Kupffer cells. Spheroids were analyzed for lipid accumulation, cytokine secretion, hepatocellular injury, and extracellular matrix remodeling using immunofluorescence, qPCR, ELISA, and LDH release assays. The model exhibited multicellular spatial organization resembling the native liver microarchitecture. Lipid accumulation was most pronounced under MASLD conditions, whereas progression to MASH was associated with stronger inflammatory and fibrosis-related changes. TNF-α showed the clearest Kupffer cell-associated increase, whereas IL-6 changes were less consistent and likely reflected the broader inflammatory response of a multicellular system. LDH release increased with disease severity and was most pronounced in MASH without evidence of a robust Kupffer cell-specific effect. Fibrotic remodeling was reflected by increased expression of fibronectin, collagen III, and procollagen I. Together, these findings establish a human multicellular *in vitro* platform that recapitulates the key disease-relevant features of MASLD/MASH under controlled conditions.

## Teaser

**Summary:** A multicellular 3D liver spheroid model recapitulates the features of MASLD and MASH, enabling translational research on lipid metabolism, immune activation, and fibrotic remodeling.

## INTRODUCTION

Metabolic dysfunction-associated steatotic liver disease (MASLD) and its progressive form, metabolic dysfunction-associated steatohepatitis (MASH), are serious and increasingly prevalent health conditions worldwide (Younossi et al., 2018). These conditions are characterized by the accumulation of fat in the liver, inflammation, and liver damage, ultimately leading to liver dysfunction and potentially liver failure if left untreated (Dulai et al., 2017; Chalasani et al., 2018; Younossi et al., 2018). Although substantial progress has been made in understanding the pathogenesis of MASLD and MASH, key mechanistic interactions and actionable therapeutic targets remain incompletely defined, underscoring the need for advanced and physiologically relevant models that can accurately mimic the pathological conditions observed in patients with MASLD and MASH. To date, MASH has mostly been studied in animal models (Hansen et al., 2017), while existing *in vitro* systems have only partially recapitulated the sequential hallmarks of steatosis, inflammation, and fibrosis that characterize disease progression (Farrell et al., 2019). However, animal models also have disadvantages; experimental animals must be fed very specific diets and require strong hepatotoxic stimuli to develop liver fibrosis in their short lifespans (Friedman, 2008).

Previously established 2D *in vitro* models have not been able to reproduce the complex interplay between different cell types in MASLD and MASH (Boeckmans et al., 2018; Cho et al., 2021). From a clinical perspective, reliance on animal systems further emphasizes the need for human-based models that better reflect patient-specific disease processes (Jahn et al., 2019). The characteristic features of MASLD and MASH involve not only hepatocytes but also non-parenchymal cells (NPCs): resident Kupffer cells (KCs), liver sinusoidal endothelial cells (LSECs), and hepatic stellate cells (HSCs) (Boeckmans et al., 2018). All these cell types are involved in the processes of steatosis, inflammation, and fibrosis and should be included in an *in vitro* model. Therefore, 3D co-cultures have been designed in recent years to study the key pathological responses of MASH (Kozyra et al., 2018; Mukherjee et al., 2019; Pingitore et al., 2019; Ströbel et al., 2021). The 3D models show key responses to hepatic injury, steatosis, inflammation, and early fibrosis. Furthermore, 3D models better represent the complexity and function of individual cell lines in the liver (Pingitore et al., 2019). The interplay between these cell types is crucial for the development of MASH, as hepatocyte dysfunction leads to lipid accumulation and oxidative stress, KCs amplify inflammation through cytokine secretion, hepatic stellate cells drive fibrotic remodeling, and endothelial cells contribute to vascular alterations that impact disease progression (Tsuchida and Friedman, 2017).

Despite recent advances in 3D liver systems, most existing models primarily recapitulate steatosis or fibrosis and lack the immune components that drive disease progression. In particular, the specific contribution of KCs to inflammatory amplification has not yet been systematically dissected in reproducible human *in vitro* liver models.

Here, we present a human four-cell 3D liver spheroid model that incorporates hepatocytes, HSCs, LSECs, and CD68-positive KCs. Specifically, we addressed the following questions: (1) can HepaRG-based multicellular spheroids recapitulate the histological and molecular hallmarks of both MASLD (steatosis) and MASH (steatohepatitis with fibrosis) under defined *in vitro* conditions? (2) Do Kupffer cells amplify inflammatory and fibrotic responses in this system? (3) Can the temporal control of disease induction [fatty acid exposure followed by a lipopolysaccharide (LPS) ‘second hit’] model sequentially induce disease progression from MASLD to MASH? This design enables the controlled temporal induction of KC-mediated inflammation on a steatotic background, bridging the gap between simplified *in vitro* systems and complex *in vivo* disease models.

## RESULTS

### Cellular organization within the 3D liver spheroids

**Fig. 1.**
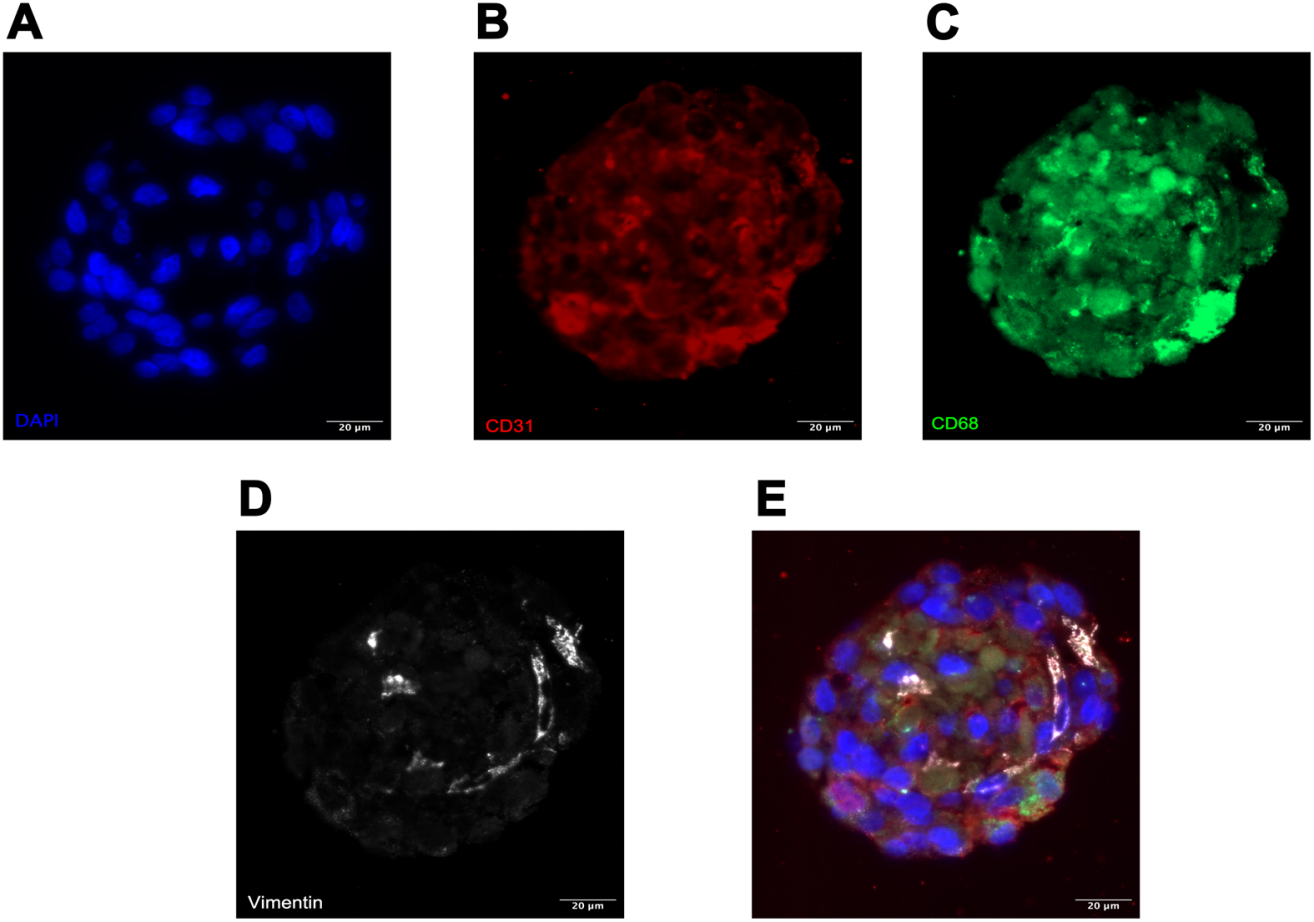
**Immunofluorescence characterization of multicellular spheroids.** Representative sections show distinct cell populations within the 3D liver spheroids on day 12. (A) DAPI (blue) stains all nuclei. (B) CD31 (red) identifies the liver sinusoidal endothelial cells (LSECs). (C) CD68 (green) highlights the KCs. (D) Vimentin (white) identifies vimentin-positive cells consistent with a hepatic stellate cells (HSCs) phenotype. (E) Merged overlay showing the spatial organization and multicellular interactions within the spheroid.

Spheroids were generated by the self-assembly of human HepaRG hepatocytes, HSCs, LSECs, and KCs in a Sphericalplate 5D microcavity platform (see Materials and Methods, ‘Spheroid generation and induction of MASLD and MASH’). The cells were allowed to compact into multicellular microtissues over 3 days before adding KCs. Immunofluorescence analysis, as shown in Fig. 1, was performed at the experimental endpoint on day 12 in spheroids maintained under control conditions, without palmitic acid (PA)/oleic acid (OA) or LPS exposure. Under standard culture conditions, the spheroids remained structurally intact and viable throughout the 19-day experimental period. The successful establishment of the 3D liver spheroid model was confirmed by immunofluorescence staining targeting specific cellular markers. Immunofluorescence analysis provided insights into the spatial arrangement of different hepatic cell types within the 3D spheroid model (Fig. 1). DAPI staining confirmed dense and structured cellular organization (Fig. 1A). The endothelial marker CD31 was predominantly observed at the spheroid periphery (Fig. 1B), suggesting the presence of CD31-positive endothelial structures there. In contrast, CD68-positive KCs (Fig. 1C) were dispersed throughout the spheroids, with some forming localized clusters within the spheroids. The mesenchymal marker vimentin (Fig. 1D) was expressed in vimentin-positive cells, consistent with a stellate cell phenotype distributed across spheroids, reflecting their role in structural support and extracellular matrix remodeling. To further support the identity of stellate cells, desmin immunofluorescence was performed as an additional HSC-specific marker, confirming the presence of desmin-positive cells within the spheroids (Fig. S2). The images illustrate the coexistence of these cellular components within the spheroid architecture, highlighting a level of organization that recapitulates important aspects of the hepatic microenvironment. The observed spatial distribution reflects the coexistence of endothelial, macrophage, and stellate cell populations, reinforcing the relevance of this model for studying liver tissue dynamics, inflammatory responses, and fibrosis *in vitro*.

### Lipid accumulation in 3D liver spheroids

**Fig. 2.**
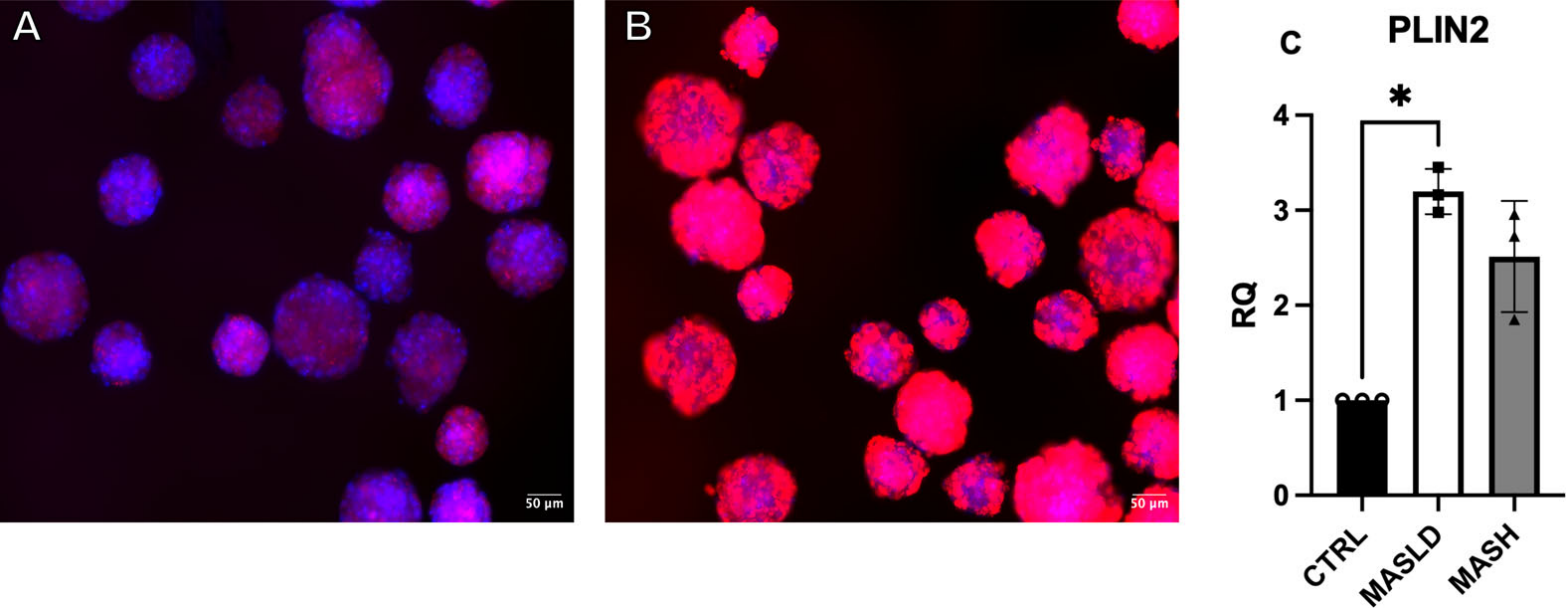
**Lipid accumulation in multicellular liver spheroids.** (A) Control spheroids exhibited minimal Nile Red staining. (B) MASLD spheroids displayed strong Nile Red staining, consistent with lipid deposition. (C) PLIN2 mRNA expression measured by qPCR at the experimental endpoint (*n*=3 biological replicates per group). The Kruskal–Wallis test followed by Dunn's post-hoc test indicated a significantly higher expression in MASLD spheroids than in controls (*P*=0.013). The difference between the MASH and control groups did not reach statistical significance (*P*=0.345).

MASLD was induced by supplementing the culture medium with PA (0.125 mM) and OA (0.25 mM) complexed with 1% fatty acid-free bovine serum albumin (BSA). For MASH induction, an acute inflammatory second hit was applied on day 5 of fatty acid treatment by adding LPS (5 μg/ml) to the MASLD medium. Neutral lipid deposition was assessed by Nile Red staining and quantification of the lipid droplet coat protein PLIN2. Nile Red fluorescence showed a stronger signal in the MASLD spheroids than in the controls (Fig. 2A,B). PLIN2 mRNA expression was determined using quantitative PCR (qPCR) at the experimental endpoint (Fig. 2C). Kruskal–Wallis testing indicated significant differences between groups (*P*=0.004). Post-hoc analysis using Dunn's test demonstrated significantly higher PLIN2 expression in MASLD spheroids than in controls (*P*=0.013), whereas the difference between MASH and controls did not reach statistical significance (*P*=0.345). A direct comparison between MASLD and MASH was not performed as the primary contrast of interest was against controls.

### Pro-inflammatory cytokine profiles

**Fig. 3.**
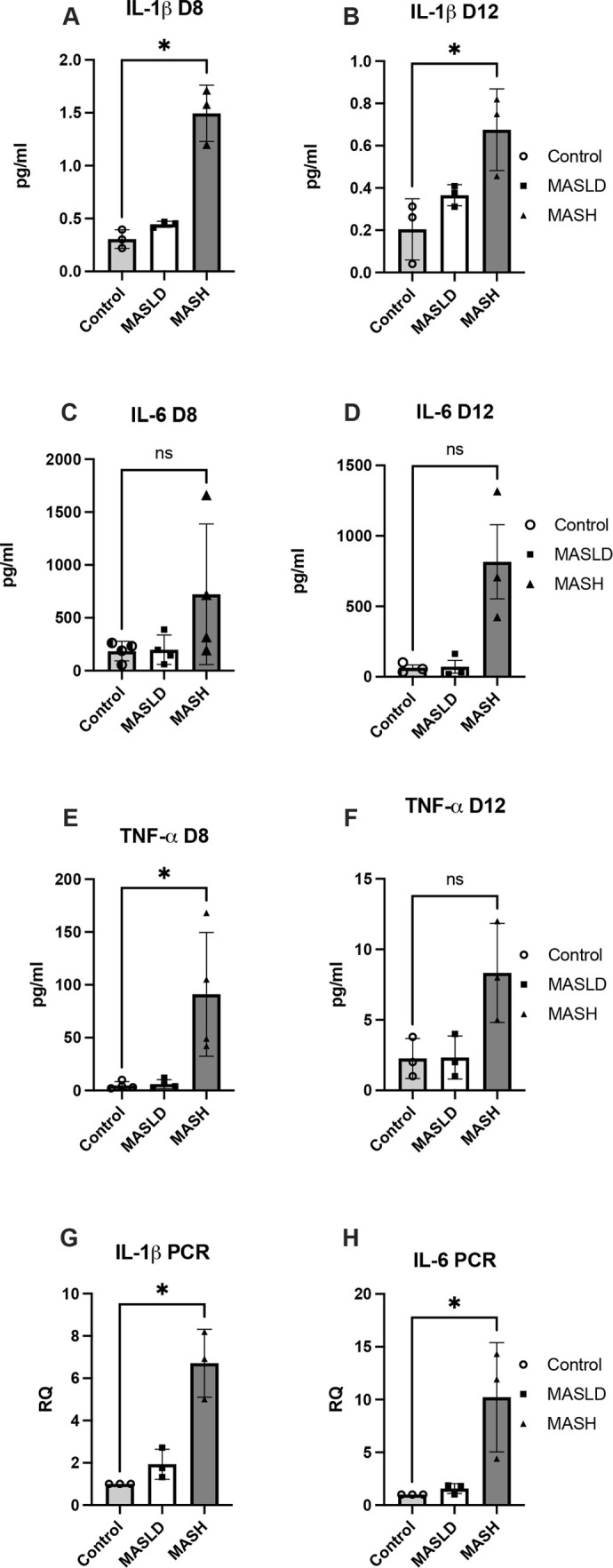
**Cytokine secretion and expression under control, MASLD, and MASH conditions.** IL-1β secretion in spheroid culture supernatants was measured by ELISA on days 8 (A) and 12 (B). IL-6 secretion was quantified by ELISA at day 8 (C) and day 12 (D). TNF-α secretion was quantified by ELISA on day 8 (E) and day 12 (F). IL-1β (G) and IL-6 (H) mRNA expression was analyzed using quantitative PCR on day 12. The control, MASLD, and MASH conditions are shown within each panel. Bars represent the mean±s.d.; dots indicate independent biological replicates. For IL-1β ELISA (A,B), *n*=3 independent biological spheroid cultures per group. For IL-6 ELISA on day 8 (C) and TNF-α ELISA on day 8 (E), *n*=4 independent biological spheroid cultures per group. For IL-6 ELISA at day 12 (D), TNF-α ELISA at day 12 (F), IL-1β qPCR (G), and IL-6 qPCR (H), *n*=3 independent biological spheroid cultures per group. Statistical analysis was performed using the Kruskal–Wallis test, followed by Dunn's multiple comparisons test with pre-specified pairwise comparisons versus the control group. IL-1β secretion showed significant overall group differences on days 8 and 12, with significantly higher levels in MASH than in control spheroids at both time points. TNF-α secretion was significantly increased in MASH compared to control spheroids on day 8, whereas IL-6 secretion did not differ significantly between groups at either time point. At the transcript level, both IL-1β and IL-6 expression were significantly increased in MASH compared to control spheroids on day 12. The exact *P*-values are provided in the Results section. Differences were considered significant at *P*<0.05.

Cytokine secretion in spheroid culture supernatants was quantified using ELISA on days 8 and 12 under control, MASLD, and MASH conditions. IL-1β release showed significant overall group differences at both time points (day 8: Kruskal–Wallis *P*=0.0036; day 12: *P*=0.0107; Fig. 3A,B). Post-hoc testing demonstrated significantly higher IL-1β secretion in MASH spheroids than in controls on day 8 (Dunn's test, adjusted *P*=0.0219) and day 12 (adjusted *P*=0.0338), whereas control and MASLD spheroids did not differ significantly at either time point.

For IL-6 secretion measured by ELISA (Fig. 3C,D), no significant overall group differences were detected on day 8 (Kruskal–Wallis *P*=0.1965) or day 12 (*P*=0.0500). Consistently, neither the control-versus-MASLD nor the control-versus-MASH comparison reached statistical significance at either time point.

TNF-α secretion, measured by ELISA, showed a time-dependent pattern (Fig. 3E,F). On day 8, Kruskal–Wallis testing demonstrated significant overall group differences (*P*=0.0087), and post-hoc analysis revealed significantly higher TNF-α levels in MASH spheroids than in controls (Dunn's test, adjusted *P*=0.0210), whereas control and MASLD spheroids did not differ significantly. By day 12, no significant overall group differences were detected (*P*=0.0571), and neither pairwise comparison reached statistical significance.

To further assess inflammatory activation at the transcriptional level, IL-1β and IL-6 mRNA expression was analyzed using quantitative PCR on day 12 (Fig. 3G,H). Both transcripts showed significant overall group differences (each Kruskal–Wallis, *P*=0.0036), with post-hoc testing demonstrating significantly higher expression in MASH spheroids than in controls (Dunn's test, adjusted *P*=0.0127 for both targets), whereas MASLD and control spheroids did not differ significantly.

### KC-associated differences in inflammatory cytokine secretion in MASLD and MASH spheroids

**Fig. 4.**
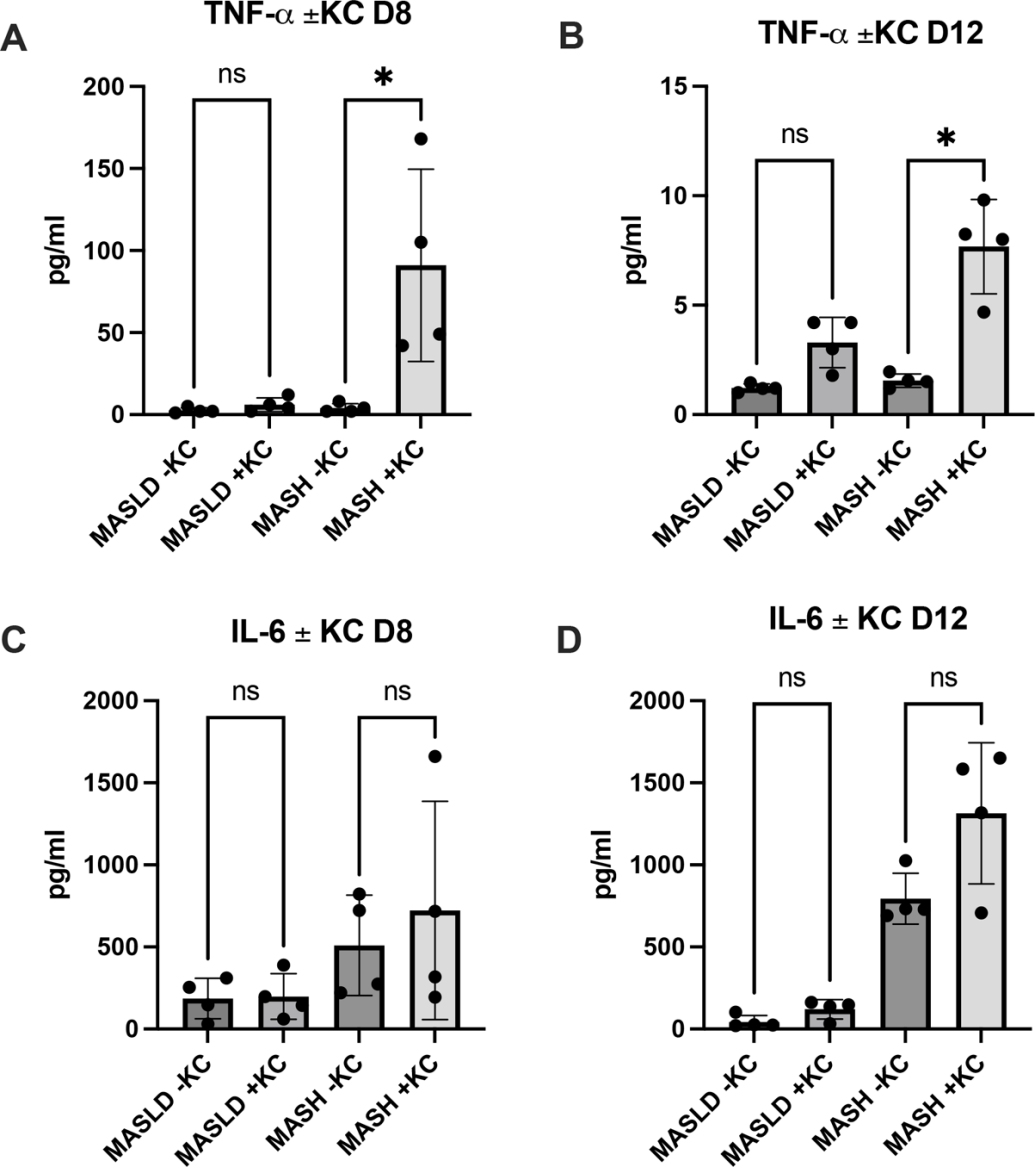
**Inflammatory cytokine secretion in MASLD and MASH spheroids cultured with or without KCs.** TNF-α concentrations in spheroid culture supernatants were measured by ELISA on days 8 (A) and 12 (B), and IL-6 concentrations were measured by ELISA on days 8 (C) and 12 (D) in MASLD and MASH spheroids cultured in the absence or presence of KCs. Each panel shows four groups: MASLD −KC, MASLD +KC, MASH −KC, and MASH +KC. Bars represent mean±s.d.; dots indicate independent biological replicates. *n*=4 independent biological spheroid cultures per group. Statistical analysis was performed using the Kruskal–Wallis test followed by Dunn's multiple comparisons test. Prespecified pairwise comparisons were performed within each disease condition to assess the effect of KC presence, comparing MASLD −KC with MASLD +KC spheroids and MASH −KC with MASH +KC spheroids. TNF-α levels were significantly higher in MASH +KC than in MASH −KC spheroids on days 8 and 12, whereas no significant difference was observed between MASLD −KC and MASLD +KC spheroids. IL-6 levels did not differ significantly between spheroids cultured without or with KCs under MASLD or MASH conditions at either time point. Exact *P*-values are reported in the Results section. Differences were considered significant at *P*<0.05.

To assess whether the presence of KCs affected cytokine secretion within the same disease condition, TNF-α and IL-6 concentrations in spheroid culture supernatants were quantified using ELISA in MASLD and MASH spheroids cultured with or without KCs. On day 8, TNF-α levels were significantly higher in MASH +KC than in matched MASH −KC spheroids (*P*=0.0043) (Fig. 4A), whereas no significant difference was observed between MASLD −KC and MASLD +KC spheroids (*P*=<0.0001). A comparable pattern was observed on day 12 (Fig. 4B), with significantly higher TNF-α levels in MASH +KC than in MASH −KC spheroids, whereas MASLD spheroids again showed no significant KC-dependent difference. In contrast, IL-6 levels did not differ significantly between spheroids cultured without and with KCs under MASLD or MASH conditions on days 8 (Fig. 4C) and 12 (Fig. 4D).

### Fibrotic remodeling

**Fig. 5.**
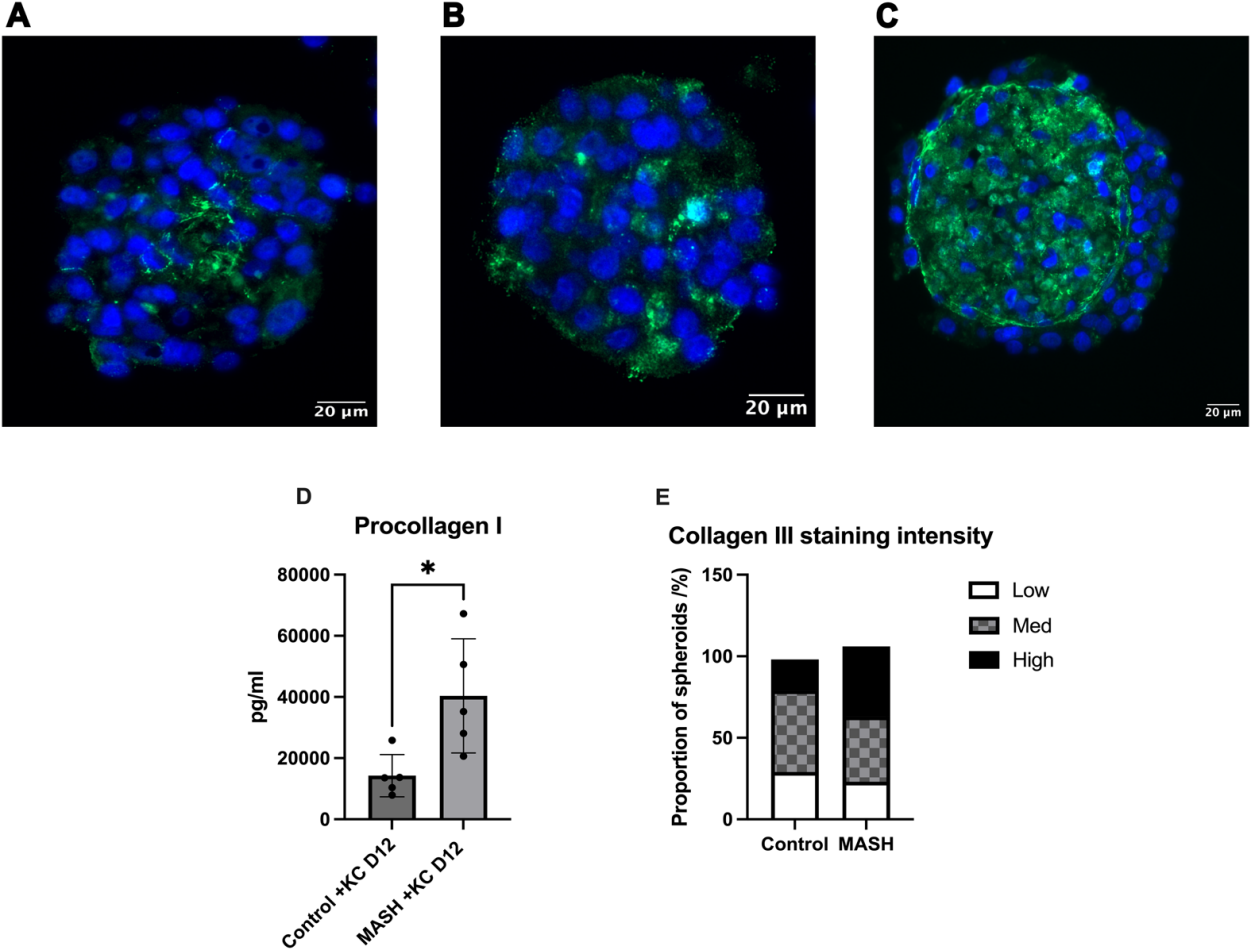
**Fibrotic remodeling of 3D liver spheroids.** (A-C) Immunofluorescence for fibronectin in control (A), MASLD (B), and MASH (C) spheroids (all panels show KC-containing spheroids). (D) Procollagen I secretion quantified by ELISA on day 12 in KC-containing spheroids was significantly higher in MASH than in controls. Bars represent mean±s.d.; dots indicate independent biological replicates (*n*=5 spheroid cultures per group, representative of two independent experiments). (E) Collagen III immunostaining intensity distributions (low, medium, high) in control and MASH spheroids from the same experimental series differed significantly (χ² test, *P*=0.0045).

Fibrotic remodeling was examined by extracellular matrix staining and quantification of collagen synthesis levels. Immunofluorescence for fibronectin showed only weak signals in control spheroids, a moderate increase in MASLD spheroids, and strong, widespread staining in MASH spheroids, consistent with the progressive matrix deposition (Fig. 5A-C).

Procollagen I secretion was quantified using ELISA on day 12. MASH spheroids showed significantly higher levels than controls [40,385±18,670 versus 14,274±6903 pg/ml; mean±s.d., *n*=5 per group; *t*(8)=2.93, *P*=0.019] (Fig. 5D).

Collagen III immunostaining revealed differences in staining intensity distribution between the control and MASH spheroids. In the controls, most spheroids exhibited low or medium staining, whereas MASH spheroids displayed an increased proportion of high staining. Chi-square analysis confirmed that the categorical distributions differed significantly between the groups (χ²=10.80, d.f.=2, *P*=0.0045; control: 29 low, 50 medium, 19 high; MASH: 23 low, 40 medium, 43 high) (Fig. 5E).

### Cytotoxicity assessment by lactate dehydrogenase release in 3D liver spheroids

**Fig. 6.**
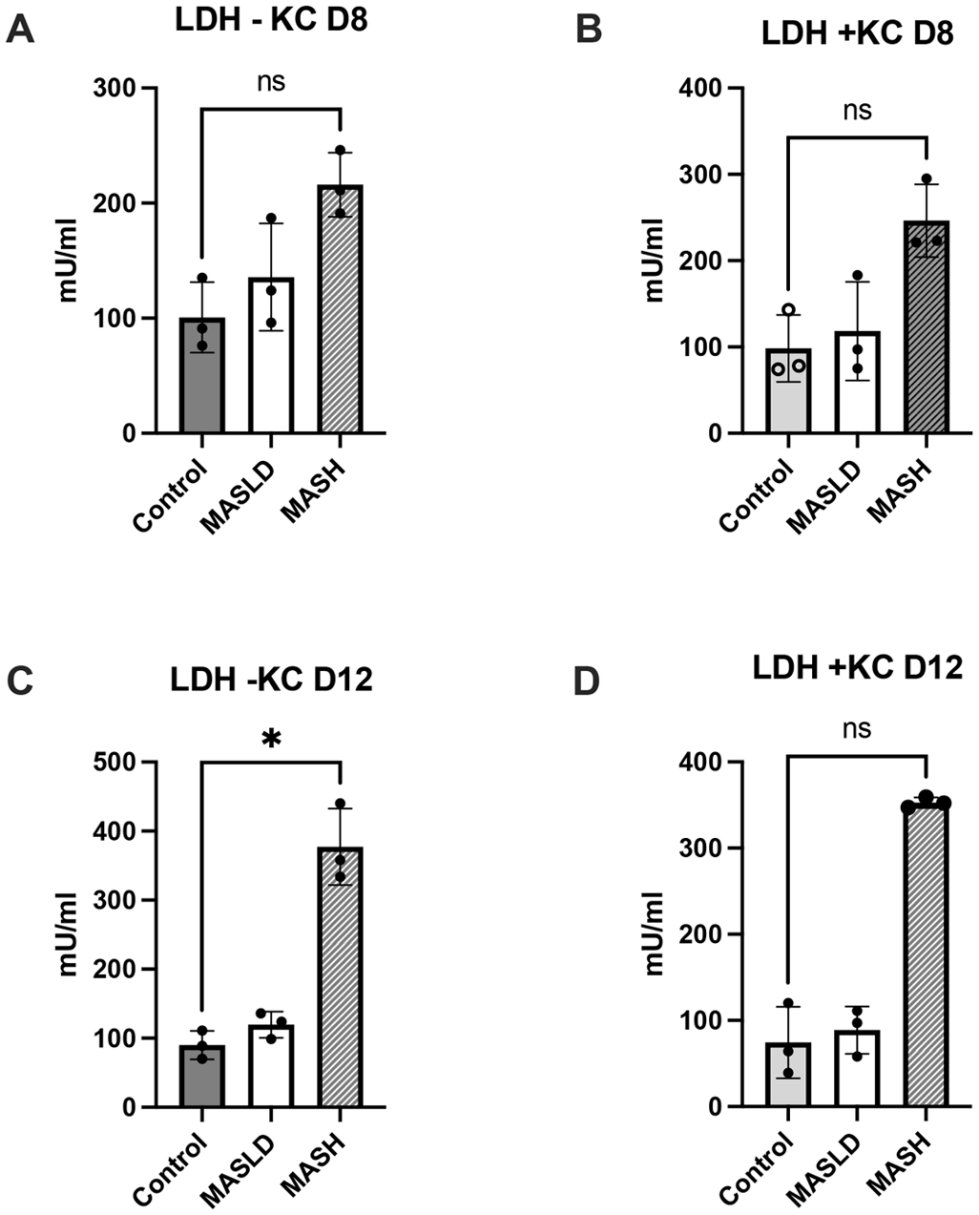
**LDH release in control, MASLD, and MASH spheroids cultured with or without KCs.** LDH was quantified as a marker of hepatocellular injury on day 8 in −KC (A) and +KC (B) spheroids and on day 12 in −KC (C) and +KC (D) spheroids. Bars represent mean±s.d.; dots indicate independent biological replicates (*n*=3 per group). Statistical analysis was performed using the Kruskal–Wallis test, followed by Dunn's multiple comparisons test. Overall group differences were detected in −KC spheroids on days 8 and 12, whereas +KC spheroids showed no significant overall differences. Pairwise post hoc testing identified a significant increase in LDH release in MASH versus control spheroids only at day 12 in the −KC condition. No pairwise comparison reached significance on day 8 after correction for multiple testing.

To assess whether Kupffer cell presence modulates hepatocellular injury beyond the disease condition itself, lactate dehydrogenase (LDH) release was measured in control, MASLD, and MASH spheroids cultured with or without KCs. LDH release patterns across conditions are shown in Fig. 6. In KC-free spheroids, Kruskal–Wallis test detected overall group differences on days 8 (*P*=0.0250) and 12 (*P*=0.0107). However, at day 8, pairwise post hoc testing did not identify significant differences between individual groups after correction for multiple comparisons, although LDH release tended to be higher in MASH than in control spheroids (Dunn's test, adjusted *P*=0.0512). At day 12, post hoc analysis revealed significantly increased LDH release in MASH compared to control spheroids (Dunn's test, adjusted *P*=0.0338). In the KC-containing spheroids, no significant overall group differences were observed on either day 8 (*P*=0.0500) or day 12 (*P*=0.0714). In addition, pre-specified within-condition comparisons at day 12 did not reveal significant differences in LDH release between −KC and +KC spheroids in either MASLD or MASH (Fig. S3). Together, these findings indicate that LDH release in this model primarily reflects disease-associated cytotoxic stress rather than a robust KC-specific effect on the liver.

## DISCUSSION

MASH is increasingly recognized as a global health concern, contributing substantially to liver-related morbidity and mortality (Arab et al., 2018; Chalasani et al., 2018). Although a range of preclinical models exist (Cole et al., 2018; Jacobs et al., 2016; Kozyra et al., 2018), most fail to reproduce the structural complexity of human liver tissue or the immune dynamics that drive inflammation and fibrotic remodeling. In this study, we established a human four-cell 3D liver spheroid model that incorporated KCs. In contrast to simplified systems focusing mainly on lipid accumulation or fibrosis, our model combines multicellular liver spheroids with temporally controlled induction of inflammation. Key comparators in the field include the InSphero 3D-hLMT system described by Mukherjee et al. (2019), which combines primary human hepatocytes with HSCs, KCs, and endothelial cells and develops a severe pro-inflammatory and pro-fibrotic phenotype in response to PA. Another important comparator is the multicellular primary human microtissue model reported by Ströbel et al. (2021), which is conceptually closer to the present approach in that MASH-like changes are induced by combined metabolic and inflammatory stimulation, including free fatty acids and a defined LPS pulse. In addition, the PNPLA3-mutant spheroid model of Pingitore et al. (2019) represents a complementary approach with a stronger emphasis on genetic susceptibility to MASLD. In contrast to these systems, the present model uses HepaRG cells as the hepatocyte source, enabling a reproducible and scalable platform while preserving relevant non-parenchymal interactions. Another distinguishing feature is the direct comparison of matched spheroids cultured with and without KCs within the same experimental framework. Future validation in primary or patient-derived hepatocyte spheroids would further strengthen the translational relevance of these findings. The resulting spheroids incorporated HepaRGs, HSCs, LSECs, and KCs, forming a microarchitecture resembling native hepatic organization. Immunofluorescence confirmed KC integration via CD68 expression, reflecting their physiological role in immune regulation during MASLD/MASH development (Kazankov et al., 2019).

Hepatocellular injury, as assessed by LDH release, exhibited a disease-dependent pattern across all conditions. In KC-free spheroids, significant group differences were detected on day 8 (Kruskal–Wallis, *P*=0.025) and day 12 (*P*=0.011), with post-hoc analysis confirming higher LDH levels in MASH than in controls on day 12 (Dunn's test, *P*=0.034). In KC-containing spheroids, group differences did not reach statistical significance at either time point (day 8: *P*=0.050; day 12: *P*=0.071), although LDH levels followed the same directional pattern. In addition, a pre-specified within-condition comparison on day 12 showed no significant difference in LDH release between MASH spheroids cultured with or without KCs (Fig. S3), indicating that KC status did not independently drive the cytotoxicity. These findings suggest that LDH in this model primarily reflects disease-associated cytotoxic stress rather than a robust KC-specific effect. This interpretation is consistent with clinical evidence linking elevated serum LDH levels to MASLD and advanced hepatic fibrosis (Zhang et al., 2025). KCs are key mediators of hepatic inflammation and respond to lipotoxic and inflammatory stimuli by releasing pro-inflammatory cytokines (Braunersreuther, 2012; Krenkel and Tacke, 2017). In the present model, cytokine secretion increased under MASH conditions, with TNF-α showing the clearest KC-associated difference between the two groups. IL-6 changes in MASH spheroids were less consistent and appeared to reflect the broader inflammatory state of the multicellular system rather than a robust KC-specific signal. Temporal variation was observed, with KC-associated TNF-α differences on both days 8 and 12, whereas IL-6 did not show a comparably robust KC-dependent pattern.

Lipid accumulation was evident in MASLD spheroids, as confirmed by Nile Red staining and PLIN2 upregulation. Under MASH conditions, PLIN2 levels were elevated compared with those in the controls but did not reach statistical significance, and Nile Red staining showed a modest reduction compared with that in MASLD spheroids. This plateau likely reflects adaptive mechanisms such as lipophagy or metabolic reprogramming and is consistent with clinical data showing that lipid deposition tends to stabilize as inflammatory processes intensify (Chalasani et al., 2018; Mejía-Guzmán et al., 2025). In contrast, inflammatory cytokines increased under MASH conditions, consistent with progressive inflammatory activation in the multicellular spheroid system (Duarte et al., 2015; Pasarín et al., 2012).

In line with the dual-hit concept of MASH pathogenesis (Buzzetti et al., 2016) and consistent with the approach used by Ströbel et al. (2021), we applied an additional inflammatory stimulus by adding LPS to FFA-treated spheroids. While fatty acid exposure alone was sufficient to induce steatosis and initiate inflammatory signaling, the addition of LPS markedly increased cytokine secretion, consistent with enhanced inflammatory activation in this multicellular system. The LPS concentration used in this study exceeded physiological serum levels but was deliberately chosen to induce a robust and reproducible inflammatory response within an experimentally tractable timeframe. Similar or higher concentrations have been used in previous studies using human liver spheroid and organoid models to accelerate MASH-like inflammation (Bell et al., 2020; Boeckmans et al., 2018; Mukherjee et al., 2019). In our protocol, LPS was applied on day 5 of fatty acid treatment, rather than on day 3, as reported by Ströbel et al. (2021). This difference in timing did not change the overall interpretation of the model but illustrated that disease kinetics can be modulated through temporal control of the inflammatory trigger.

Fibrotic remodeling was another prominent feature of this model. Immunofluorescence for fibronectin showed weak staining in control spheroids, a moderate increase in MASLD, and widespread strong staining in MASH spheroids, consistent with increased extracellular matrix deposition in MASH spheroids. Although some extracellular matrix deposition was also present in the control spheroids, this likely reflects the structural requirements for spheroid stability (Song et al., 2016). Collagen III immunohistochemistry also revealed a significant shift toward higher staining intensity in MASH spheroids compared with controls, while ELISA confirmed increased secretion of procollagen I. These results align with clinical observations, where the fibrosis stage is the strongest predictor of liver-related outcomes in MASH (Dulai et al., 2017; Vilar-Gomez et al., 2018). Comparable findings have been reported in other 3D models: Mukherjee et al. (2019) described bridging fibrosis-like collagen deposition after palmitate treatment (Mukherjee et al., 2019), Pingitore et al. (2019) demonstrated collagen induction in PNPLA3-mutant spheroids (Pingitore et al., 2019), and Ströbel et al. (2021) reported strong fibrotic signatures, including procollagen I and TIMP-1, in their FFA+LPS-induced model (Ströbel et al., 2021). Our data add to these observations by showing that fibrotic remodeling can be captured in HepaRG-based multicellular spheroids, supporting the use of this system for future studies of early anti-fibrotic interventions (Cho et al., 2021).

The present study has several limitations. Spheroids were generated by stochastic self-assembly, which does not allow for precise control over the cellular composition at the individual spheroid level. Hepatocyte functionality was not formally validated by albumin secretion or HNF4α immunostaining; however, differentiated HepaRG cells are well-established to retain key hepatocyte functions (Aninat et al., 2006; Bronsard et al., 2024). Stellate cell identity was supported by both vimentin and desmin expression (Fig. S2), although further markers such as LRAT or PDGFRβ were not assessed in this study. Endothelial structures were identified by peripheral CD31 staining but not formally quantified, and high-resolution imaging of KC distribution was not performed. Instead, KC integration was confirmed at the transcriptional level (Fig. S1). Adaptive immune cells are absent, the culture duration is limited, and wide-field fluorescence microscopy is used throughout, restricting high-resolution spatial analysis. As the model is based on HepaRG cells, validation in primary or patient-derived systems is important. The effects of innate immune recognition between donor-mismatched populations, including possible SIRPα/CD47-related interactions, cannot be excluded. Despite the limited number of independent spheroid cultures per assay, the principal findings were supported by concordant directional changes across complementary readouts, including cytokine secretion, inflammatory gene expression, extracellular matrix remodeling, and cytotoxicity, and were therefore interpreted conservatively. Future studies should address these aspects by extending the culture duration, refining imaging approaches, and incorporating primary cell sources and additional immune populations.

By incorporating KCs and enabling temporal control of inflammatory induction, this model provides a physiologically relevant and experimentally tractable platform for studying disease-relevant immunometabolic interactions associated with MASH progression. A particular strength of the present study is the direct comparison of matched spheroid conditions in the presence and absence of KCs in the culture medium. This may be useful in the context of metabolic liver disease, where the contribution of hepatic macrophages to inflammatory amplification and fibrotic remodeling remains of considerable interest. Although pharmacological validation was not the primary objective of the present study, the system may provide a useful basis for future studies on immunomodulatory and anti-fibrotic strategies in a controlled human *in vitro* setting.

### Conclusion

In summary, we established a 3D human liver spheroid model that captures the key pathological features of MASLD/MASH, including steatosis, inflammatory activation, hepatocellular injury, and fibrotic remodeling. An important feature of this system is the inclusion of KCs, which allows inflammatory readouts to be examined in a multicellular setting under defined disease conditions. By bridging the gap between reductionist 2D cultures and more complex *in vivo* models, this platform provides a physiologically relevant and experimentally accessible framework for translational research. Future studies should focus on incorporating primary or patient-derived hepatocytes and extending the culture duration to better reflect long-term disease trajectories, thereby further strengthening its translational relevance.

## MATERIALS AND METHODS

### Culturing of HepaRG, HSCs, KCs and LSECs

All cell lines were cultivated at 37°C in 5% CO_2_ and 95% humidity. HepaRG cells (human hepatocyte cell line) (Thermo Fisher Scientific, Waltham, MA, USA) were cultured in William's medium (PAN Biotech, Aidenbach, Germany) with 10% HyClone FetalClone 2 serum (Cytiva, Marlborough, MA, USA), 1% L-glutamine solution (Sigma-Aldrich, St. Louis, MO, USA), 1% penicillin-streptomycin (Sigma–Aldrich), 12.8 U of human insulin (Lilly, Indianapolis, IN, USA), and 53 μl of hydrocortisone (Pfizer, New York, NY, USA) and passaged every 3-4 days with a seeding density of 1.7×10^5^ cells/cm^2^ in collagen-coated flasks (Sigma-Aldrich). Differentiation of HepaRG cells was performed by cultivation in William's medium for 2 weeks, followed by cultivation in William's medium with 1.8% DMSO (Sigma-Aldrich) for another 2 weeks. Primary human HSCs were obtained from iXCells Biotechnologies (San Diego, CA, USA) using the recommended medium. After thawing, HSCs were cultured in Petri dishes until they reached 90% confluence. Primary human KCs (Lonza, Walkersville, MD, USA), which are primary cells that do not proliferate *in vitro*, were thawed and initially resuspended in the KuGM™ Kupffer Cell Culture Bullet Kit™ (Lonza). The cells were then counted and transferred to a Phenol Red- and hydrocortisone-free medium, which was used for all subsequent experiments. Primary human LSECs (Innoprot, Derio, Spain) were seeded at a density of 5×10^3^ cells/cm^2^ in fibronectin-coated (Innoprot) flasks in Endothelial Cell Medium (Innoprot) until 90% confluence was reached, at which point passaging was performed using the primary cell detach kit (Innoprot).

### Spheroid generation and induction of MASLD and MASH

**Fig. 7.**
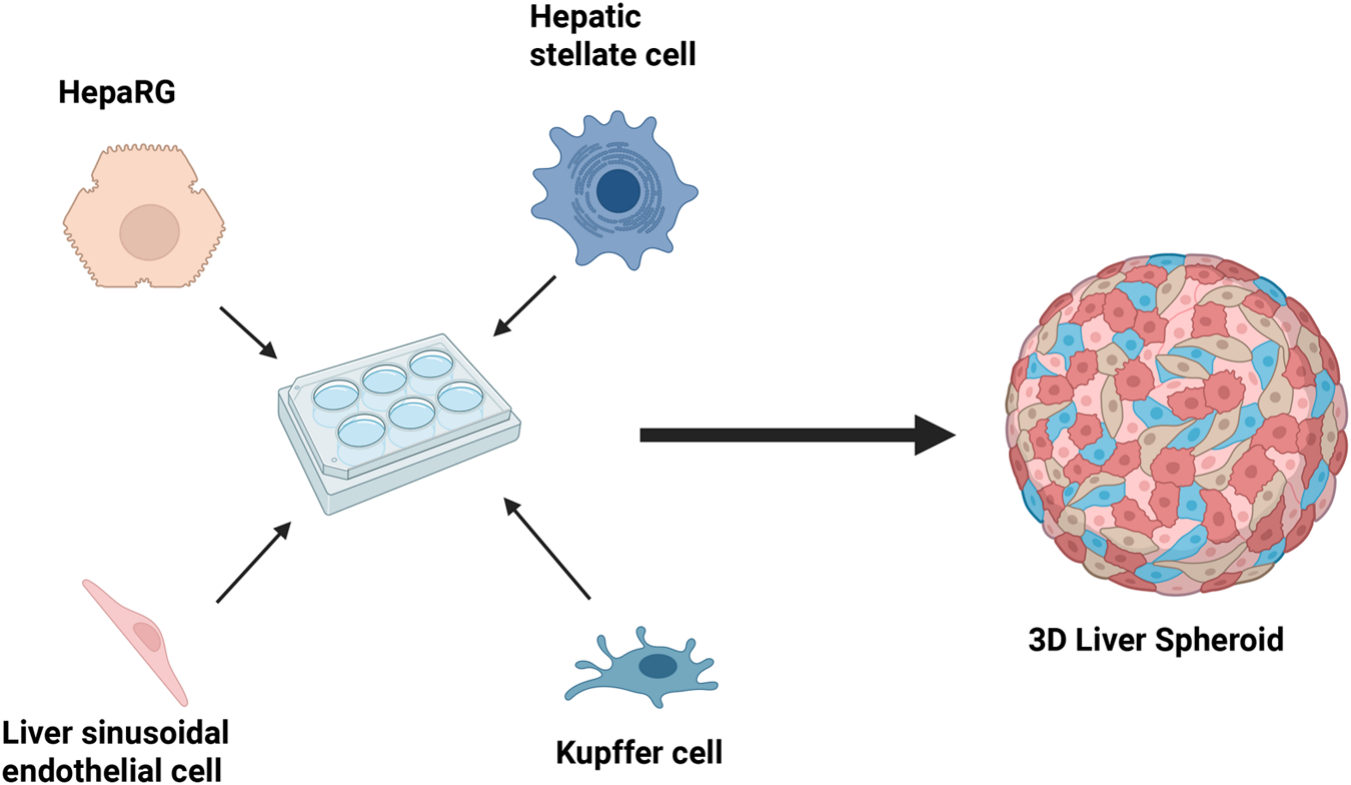
**Schematic overview of 3D liver spheroid generation.** Human HepaRG hepatocytes, HSCs, KCs, and LSECs were combined in defined ratios and seeded into a Sphericalplate 5D. Within this microcavity platform, cells self-assemble into uniform multicellular spheroids. The illustration depicts the cell types and their arrangement into a structured liver-like microtissue that recapitulates aspects of the hepatic microarchitecture. Created in BioRender by Bogovic, N. (2025). https://BioRender.com/8zmhwub. This figure was sublicensed under CC-BY 4.0 terms.

Spheroids of the four cell lines were cultivated in a Sphericalplate 5D (Heidolph, Schwabach, Germany) according to the manufacturer's instructions (Kugelmaier SOP, SP5D6). A schematic overview of spheroid generation is shown in Fig. 7. A total of 350 cells per microwell were seeded, corresponding to approximately 2.6×10^5^ cells per well (24-well format, 750 microwells/well). NPCs include HSCs, LSECs, and KCs. Spheroids were seeded at a ratio of 80% HepaRG hepatocytes to 20% NPCs. Owing to the multicellular format of the Sphericalplate 5D (750 spheroids/well), cell distribution cannot be controlled at the level of individual spheroids. Across independent preparations, the average NPC distribution was approximately 6.5% HSCs, 6.5% LSECs, and 9.6% KCs within the NPC fraction. Following one medium exchange, KCs were added 2 days prior to fatty acid stimulation using a medium without Phenol Red and hydrocortisone.

### Fatty acid preparation and MASH induction

OA (Sigma-Aldrich, St. Louis, MO, USA) 01008-25G) and PA (Sigma-Aldrich, cat. P0500-10G) were dissolved in 2-propanol (Sigma-Aldrich, cat. I9516-500 ml, purity ≥99.5%). A mixed stock solution was prepared by combining 1 ml of PA (50 mM) and 2 ml of OA (50 mM), resulting in a solution containing 16.6 mM PA and 33.3 mM OA. For treatment, this mixture was added to the culture medium at 7.5 μl/ml, yielding final concentrations of 0.125 mM PA and 0.25 mM OA.

Fatty acid-free BSA (Carl Roth, cat. 0052.3) was added to the fatty acid medium at a final concentration of 1%. Control spheroids received vehicle (2-propanol 7.5 μl/ml; 0.746% v/v) supplemented with 1% fatty acid-free BSA, matching the solvent content of the FA-treated conditions.

**Fig. 8.**
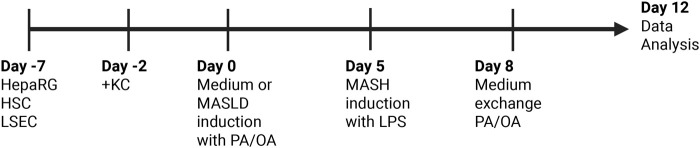
**Experimental timeline for the spheroid treatment.** Spheroids were cultured under conditions corresponding to healthy controls, MASLD, and MASH. The schematic summarizes the timing of fatty acid exposure, inflammatory stimulation, and endpoint analysis. Created in BioRender by Bogovic, N. (2026). https://BioRender.com/lr7g76d. This figure was sublicensed under CC-BY 4.0 terms.

For MASH induction, on day 5 of fatty acid treatment, an acute inflammatory ‘second hit’ was applied by adding LPS (5 μg/ml) to the MASLD medium. After 19 days of total cultivation, the control, MASLD and MASH groups were harvested. Endpoint analyses were performed on days 8 and 12 of the disease-inducing treatment. Day 8 was selected to capture early phase responses, occurring 3 days after the LPS ‘second hit’ in MASH conditions, while day 12 was chosen to assess sustained or chronic effects approximately 1 week after the inflammatory stimulus. These two time points were intentionally selected to contrast early and more established disease-related changes rather than consecutive days to maximize the mechanistic contrast between the early and late response phases. The experimental treatment timeline is summarized in Fig. 8. Spheroids remained structurally intact and showed no evidence of spontaneous disintegration throughout the 19-day culture period under all tested conditions.

### ELISA

The levels of human IL-6 (cat. DY206-05, R&D Systems, Minneapolis, MN, USA), TNF-α (cat. DY210-05, R&D), IL-1β (cat. DY201-05, R&D) and ProCollagen I (cat. DY6220-05, R&D) were measured in the microtissue supernatants collected on days 8 and 12. Briefly, supernatants from four microtissues were pooled per culture and processed according to the manufacturer's instructions. The plates were read using a microplate reader at the specified wavelength.

### mRNA analysis

RNA was extracted using the RNeasy Plus Micro Kit (Qiagen, Hilden, Germany) and reverse-transcribed using the High-Capacity cDNA Reverse Transcription Kit (Thermo Fisher Scientific) according to the manufacturer's protocols. qPCR was performed using the QuantiNova SYBR Green PCR Kit (Qiagen, cat. no. 208056). Gene expression was assessed using Qiagen RT² qPCR Primer Assays (cat. no. 330001): IL1-β (GeneGlobe ID PPH00171C), IL6 (GeneGlobe ID PPH00560C), PLIN2 (GeneGlobe ID PPH02583A), and ACTB as a housekeeping gene (GeneGlobe ID PPH00073G). PCR was performed under standard QuantiNova cycling conditions. Relative gene expression levels were calculated using the ΔΔCt method, with control spheroids as a reference.

### Cell viability

Cell damage was assessed using the LDH-Glo™ Cytotoxicity Assay (Promega, Madison, WI, USA, cat. J2380). The supernatants were diluted 1:25 before analysis.

### Immunostaining

All images were captured using an Axio Observer Z1 microscope (Zeiss, Oberkochen, Germany). For Nile Red staining, a 1 mg/ml stock solution (Thermo Fisher Scientific, cat. 415711000) was prepared in acetone and stored at−20°C. The working solution was freshly generated by diluting 14.3 μl of Nile Red stock in 10 ml of PBS and supplemented with 4 μl of DAPI (5 mg/ml stock in H₂O, Sigma-Aldrich, cat. 10236276001) to yield a final concentration of 1:700 for Nile Red and 1:2500 for DAPI. Staining was performed on 4% paraformaldehyde-fixed spheroids that were placed on histological slides.

Single immunostaining: For immunofluorescence and chromogenic staining, antigen retrieval was performed using citrate buffer (pH 6.0, 10×, Zytomed, Cat. ZUC028-500) or Tris/EDTA buffer (pH 9.0, 10×, Zytomed, cat. ZUC029-500), depending on the requirements of the primary antibody. Endogenous peroxidases and phosphatases were blocked using BLOXALL (Vector Laboratories, Newark, CA, USA). Autofluorescence was minimized by incubating with TrueBlack (Biotrend, Cologne, Germany). The samples were then blocked with 10% goat or donkey serum (Sigma-Aldrich) in PBS before overnight incubation with primary antibodies at 4°C. The primary antibodies used were collagen III (1:50; Abcam, cat. ab7778) and fibronectin (1:100; Abcam, cat. ab2413). Negative controls were incubated overnight at 4°C with PBS containing 10% serum. After an additional blocking step, the samples were incubated for 1 h with secondary antibodies (Thermo Fisher Scientific), and staining was performed using DAPI (1:1000).

### Chromogenic staining

Chromogenic staining for collagen III (1:50; Abcam, cat. no. ab7778) involved dehydration through an ethanol series and antigen retrieval, followed by overnight incubation at 4°C with the primary antibody. Detection was performed using the ZytoChem Plus Kit (Zytomed, Berlin, Germany), visualized with a DAB Substrate Kit (Zytomed), and counterstained with Mayer's hematoxylin (Sigma-Aldrich).

### Triple immunostaining

Samples were subjected to a descending ethanol series, followed by antigen retrieval in citrate buffer (Zytomed). Endogenous peroxidases and phosphatases were blocked using BLOXALL (Vector Laboratories), and autofluorescence reduction was performed using TrueBlack (Biotrend). Blocking was performed using IHC/ICC Blocking Buffer (Thermo Fisher Scientific) or 3% BSA (specifically for CD68 staining). Primary antibodies diluted in PBS containing 10% serum (Sigma-Aldrich/Bio-Rad) were incubated overnight at 4°C. The antibodies used included desmin (1:1000; Abcam, cat. ab32362), vimentin (1:200; Abcam, cat. ab92547) for stellate cell identification, and CD31 (1:50; Thermo Fisher Scientific, cat. MA5-13188) and CD68 (1:200; Thermo Fisher Scientific, cat. MA5-13324). Negative controls were incubated overnight at 4°C with PBS containing 10% serum. After a subsequent protein-blocking step, secondary antibodies (Thermo Fisher Scientific) were applied for 1 h. Triple staining for different cell types was performed sequentially over consecutive days, and the nuclei were visualized with DAPI (1:1000).

### Statistics

Biological replicates were defined as independent spheroid cultures prepared from separate cell batches, whereas technical replicates referred to repeated measurements from the same culture or pooled spheroids. For ELISAs, supernatants from four spheroids were pooled per culture and analyzed in duplicate. The *n*-values reported in the figure legends refer to the number of independent cultures. For qPCR, RNA was isolated from independent spheroid preparations (*n*=3 per condition), each analyzed in technical duplicates. LDH assays were performed on independent cultures (*n*=3 per condition) with measurements in technical duplicates. Immunostaining experiments were performed on multiple spheroids per culture, and representative images are shown.

Statistical analyses were performed using GraphPad Prism version 10.3.1 (GraphPad Software, San Diego, CA, USA). Data are presented as mean±s.d. or median, as appropriate. Given the small sample sizes in this study (*n*=3-4 per group), non-parametric tests were used as defaults. Group differences were analyzed using the Kruskal–Wallis test, followed by Dunn's multiple comparisons test. The Mann–Whitney test was used for pairwise comparisons. A chi-square test was used for categorical outcomes (collagen III staining intensity). Procollagen I secretion (*n*=5 per group) was compared between the two groups using an unpaired two-tailed Student's *t*-test. Statistical significance was set at *P*<0.05.

The sample sizes were based on the availability of independent spheroid cultures. No formal power calculations were performed. No data were excluded from the analysis. Randomization was not applicable because the spheroids were seeded and allocated according to the plate design. The investigators were not blinded during the data collection or analysis. Statistical analyses were performed as described above.

## Supplementary Material

10.1242/biolopen.062552_sup1 Supplementary information
