# Supplementary material for: Glimpse into the role of Kupffer cells in a spheroid model of metabolic dysfunction-associated steatohepatitis (MASH)
Source: Biol Open. 2026 Jun 3;15(6):bio062552. doi: 10.1242/bio.062552 (PMC13312915; doi:10.1242/bio.062552)
Supplement: Supplementary information [file biolopen-15-062552-s1.pdf]

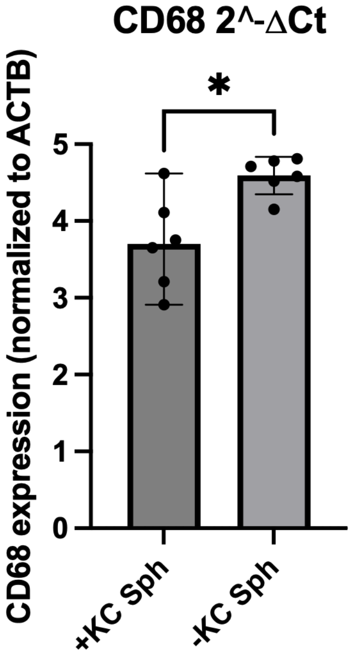

Fig. S1. CD68 mRNA expression in KC-containing and KC-free spheroids. CD68 ΔCt values (normalized to ACTB) from two independent experiments (n = 6 per group). Lower ΔCt indicates higher mRNA expression. Bars show median; dots indicate individual biological replicates. Mann-Whitney test, $p = 0.015$.

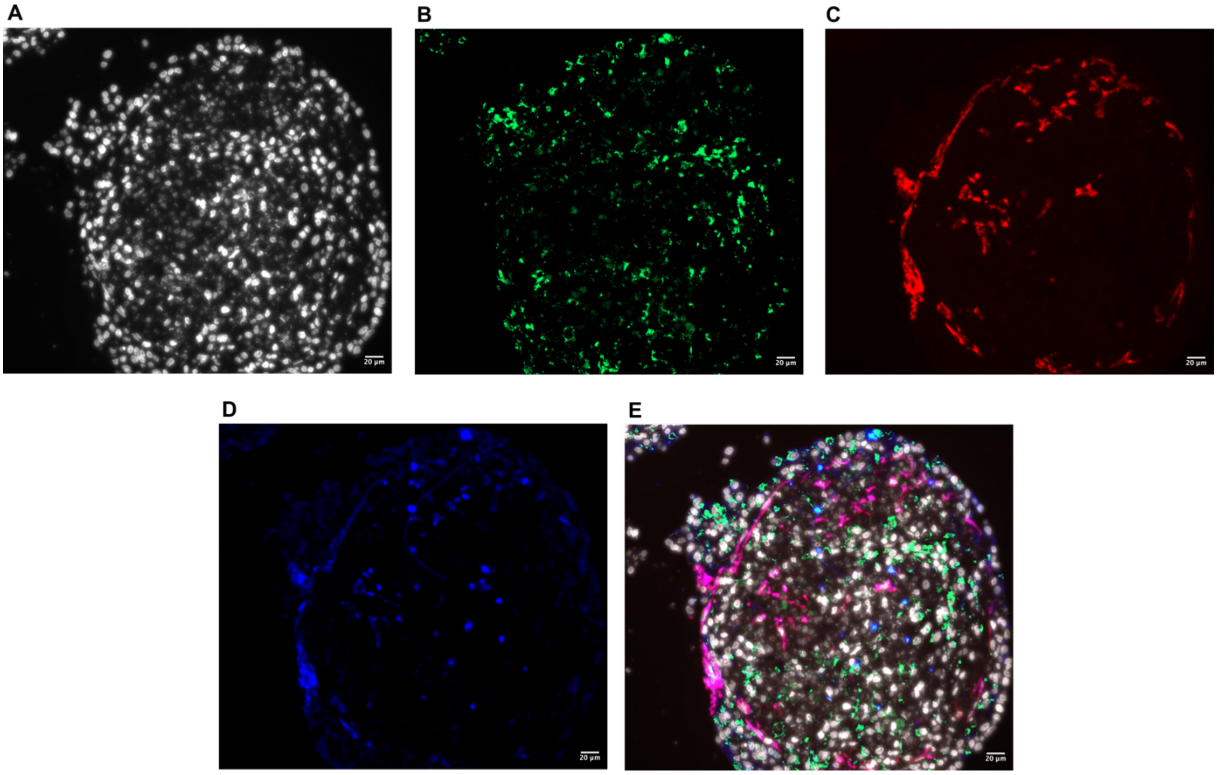

Biology Open • Supplementary information

Fig. S2. Additional immunofluorescence characterization of 3D liver spheroids using desmin as a secondary marker for hepatic stellate cells. Additional immunofluorescence characterization of 3D liver spheroids using desmin as a secondary marker for hepatic stellate cells. Representative images of a MASH spheroid showing (A) DAPI (white), labeling all nuclei, (B) CD68 (green), labeling Kupffer cells, (C) desmin (red), labeling cells consistent with a hepatic stellate cell phenotype, (D) CD31 (blue), labeling liver sinusoidal endothelial cells, and (E) merged overlay. Scale bars: 20 µm.

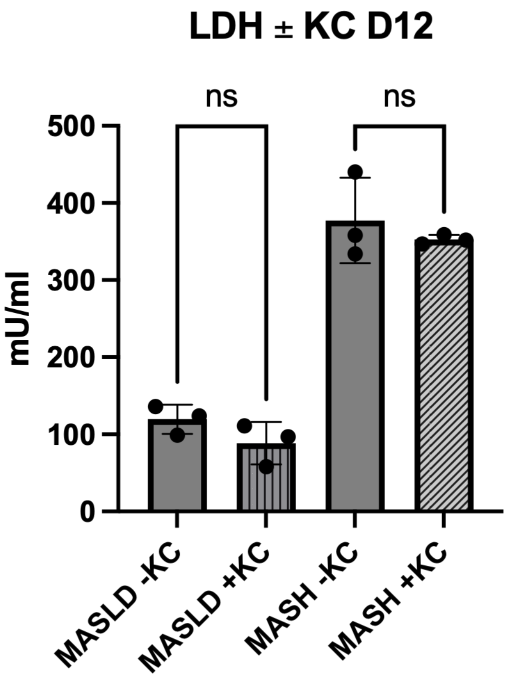

Fig. S3. Direct comparison of LDH release in MASLD and MASH spheroids cultured with or without Kupffer cells at day 12. LDH levels were measured as a marker of hepatocellular injury at day 12 in MASLD and MASH spheroids cultured in the absence (−KC) or presence (+KC) of Kupffer cells. Bars represent mean ± s.d.; dots indicate independent biological replicates. n=3 independent biological spheroid cultures per group. Statistical analysis was performed using the Kruskal–Wallis test followed by Dunn's multiple comparisons test. Pre-specified pairwise comparisons assessed the effect of Kupffer cell presence within each disease condition, comparing MASLD −KC with MASLD +KC spheroids and MASH −KC with MASH +KC spheroids. No significant differences were observed in either comparison.

Biology Open • Supplementary information
